# Application and validation of the machine learning-based multimodal radiomics model for preoperative prediction of lateral lymph node metastasis in papillary thyroid carcinoma

**DOI:** 10.3389/fendo.2025.1618902

**Published:** 2025-08-19

**Authors:** Jia-Wei Feng, Yu-Xin Yang, Rong-Jie Qin, Shui-Qing Liu, An-Cheng Qin, Yong Jiang

**Affiliations:** ^1^ Department of Thyroid Surgery, The Third Affiliated Hospital of Soochow University, Changzhou First People’s Hospital, Changzhou, Jiangsu, China; ^2^ The Second Clinical Medical School of Nanjing Medical University, Nanjing, Jiangsu, China; ^3^ Department of Ultrasound, The Third Affiliated Hospital of Soochow University, Changzhou First People’s Hospital, Changzhou, Jiangsu, China; ^4^ Department of Thyroid Surgery, Suzhou Municipal Hospital, The Affiliated Suzhou Hospital of Nanjing Medical University, Suzhou, Jiangsu, China

**Keywords:** papillary thyroid carcinoma, lateral lymph node metastasis, radiomics, multimodal prediction, machine learning

## Abstract

**Background:**

Papillary thyroid carcinoma (PTC) frequently develops lateral lymph node metastasis (LLNM) in 12.6%-32.8% of patients, increasing recurrence risk and mortality. Current diagnostic methods show significant limitations, with occult LLNM rates of 41.0%-51.7% requiring secondary surgeries. This study aims to develop and validate a multimodal prediction model integrating clinical, ultrasound, and CT radiomics features for accurate preoperative LLNM prediction in PTC patients.

**Methods:**

Clinical data, ultrasound and CT images from 799 PTC patients were retrospectively analyzed (524 training, 225 internal validation, 50 external validation). Clinical features were selected through logistic regression after collinearity analysis. A total of 874 ultrasound radiomics features and 1433 CT radiomics features were extracted and selected using LASSO regression. Four machine learning models were constructed and compared, with model interpretability explored using SHAP and LIME analyses.

**Results:**

Logistic regression identified five independent clinical risk factors: maximum tumor diameter, multiple lesions, upper pole location, decreased monocyte count, and lower lymphocyte-to-monocyte ratio (LMR). LASSO regression selected 4 key ultrasound features and 11 key CT features. The Gradient Boosting Machine (GBM) model demonstrated superior performance, with areas under the curve of 0.973, 0.803, and 0.975, and accuracies of 0.914, 0.725, and 0.900 in the training, internal validation, and external validation sets respectively. Decision curve analysis confirmed the GBM model’s highest net clinical benefit. SHAP analysis identified LMR as the most important predictor.

**Conclusion:**

The GBM-based multimodal prediction model accurately predicts LLNM in PTC patients preoperatively. This non-invasive, interpretable tool enables individualized risk assessment, potentially reducing missed metastases requiring secondary surgery, thereby supporting precise treatment decisions in PTC management.

## Introduction

Papillary thyroid carcinoma (PTC) is the most common thyroid malignancy, with lateral lymph node metastasis (LLNM) occurring in approximately 12.6%-32.8% of patients and significantly increasing recurrence risk and mortality (1).

The 2015 American Thyroid Association guidelines recommend that lateral neck dissection be reserved for patients with preoperative evidence of LLNM rather than performed routinely as a prophylactic measure (2). However, current diagnostic methods have substantial limitations. Ultrasound, while widely accessible, has insufficient sensitivity for detecting small metastases (3). Fine-needle aspiration cytology (FNAC) improves accuracy but is restricted to visibly suspicious nodes, leaving many occult metastases undetected. Studies report occult LLNM rates of 41.0%-51.7% (4, 5), representing a significant clinical challenge as undetected metastases can lead to disease persistence requiring secondary surgical intervention.

Radiomics has emerged as a promising approach to address these challenges. By employing high-throughput computational methods to extract quantitative features from medical images, radiomics can reveal biological behaviors invisible to the naked eye (6). These features—including morphological, statistical, textural, and wavelet-transformed parameters—provide comprehensive characterization of tumor heterogeneity and microenvironment, potentially offering valuable insights into metastatic potential.

Multimodal approaches that integrate clinical features with different imaging modalities offer superior predictive performance by leveraging complementary advantages of various data sources. Machine learning algorithms, including Random Forest (RF), Gradient Boosting Machine (GBM), Support Vector Machine (SVM), and K-Nearest Neighbors (KNN), can effectively analyze these high-dimensional, heterogeneous datasets to generate accurate predictive models (7).

This study aims to develop and validate a multimodal prediction model integrating preoperative clinical characteristics with ultrasound and computed tomography (CT) radiomics features to detect LLNM in PTC patients. Using machine learning and advanced model interpretation techniques, we seek to provide a non-invasive, accurate, and interpretable tool for personalized treatment planning. This approach aims to guide lateral neck dissection decisions, reduce unnecessary second surgeries, and improve surgical management of PTC patients.

We present this study in accordance with the TRIPOD (Transparent Reporting of a multivariable prediction model for Individual Prognosis Or Diagnosis) reporting guideline to ensure transparent and complete reporting of our prediction model development and validation.

## Methods

### Patients and study design

This retrospective study was approved by the Ethics Committees of Changzhou First People’s Hospital and Suzhou Municipal Hospital. Clinical data, ultrasound, and CT images were collected from thyroid cancer patients treated at these hospitals between January 2022 and June 2024. Inclusion criteria: (1) pathologically confirmed primary classic PTC; (2) preoperative ultrasound and CT meeting analysis standards; (3) complete clinical data; (4) no prior thyroid surgery/ablation; (5) patients with PTC and/or concurrent benign thyroid conditions (nodular goiter, Hashimoto’s thyroiditis). Exclusion criteria: (1) non-classic PTC or other thyroid subtypes; (2) prior thyroid surgery/ablation; (3) history of head/neck cancer or familial cancer; (4) poor imaging quality; (5) incomplete clinical data; (6) non-curative surgery with persistent disease. A total of 799 PTC patients were enrolled, with data allocation as follows: Changzhou First People’s Hospital (training group, n=524; internal validation group, n=225) and Suzhou Municipal Hospital (external validation group, n=50).

### Clinical data collection

Body mass index (BMI) was calculated as weight (kg)/height² (m²). Patients were classified as normal weight, overweight, or obese based on World Health Organization guidelines (8). Hashimoto’s thyroiditis was diagnosed based on elevated antibodies or ultrasound findings. Extrathyroidal extension (ETE) was defined as >25% tumor contact with the thyroid capsule (9). The largest lesion was used for pathological evaluation in multifocal cases. All diagnoses and lymph node statuses were confirmed pathologically. Preoperative laboratory tests included white blood cell count, platelet count, neutrophil count, lymphocyte count, monocyte count, and thyroid function, as well as inflammatory indices (lymphocyte-to-monocyte ratio (LMR), neutrophil-to-lymphocyte ratio, platelet-to-lymphocyte ratio, systemic immune-inflammation index).

### Surgical procedures

All surgical procedures were performed by experienced thyroid surgeons following standardized protocols. Thyroidectomy procedures included: (1) Total thyroidectomy: complete removal of both thyroid lobes and isthmus; (2) Thyroid lobectomy: unilateral thyroid lobe removal with isthmus. Central lymph node dissection was performed in all patients, involving systematic removal of compartment VI lymph nodes. For patients with preoperative evidence of LLNM confirmed by fine-needle aspiration cytology, therapeutic lateral lymph node dissection was performed, involving systematic removal of levels II-V lymph nodes. A total of 102 patients underwent lateral neck dissection of levels II-V: 92 patients at Changzhou First People’s Hospital (61 training group + 26 internal validation group with confirmed LLNM, plus 5 patients with suspected but pathologically negative LLNM) and 10 patients at Suzhou Municipal Hospital with confirmed LLNM.

### Preoperative imaging and diagnostic workflow

CT scans were performed with a Siemens Somatom Definition Flash dual-source CT scanner. Patients were positioned supine with slight neck hyperextension. The scanning range extended from the hyoid bone to the sternal manubrium, and if needed, to the aortic arch. Parameters included 120 kV, 200 mAs, 1.0 mm slice thickness, pitch 1.0, and a 200 mm × 200 mm field of view. Contrast-enhanced scans were performed using Iohexol (350 mg/ml iodine concentration), with dual-phase enhancement for arterial and venous phase imaging. Ultrasound was conducted using Philips iU22/EPIQ 5 or GE LOGIQ E9 systems. Experienced physicians obtained high-resolution tumor images and Doppler flow images, which were stored in DICOM format. Lymph nodes with suspicious features (e.g., round shape, absent echogenic hilum, microcalcifications) were classified as ultrasound-suspected LLNM. FNAC was then performed to confirm the histopathologic diagnosis of suspicious lateral lymph nodes. For patients with clinically suspicious LLNM confirmed by FNAC, thyroidectomy plus central neck dissection and therapeutic lateral neck dissection were performed.

### Image analysis and radiomics feature extraction

To standardize image analysis and ensure cross-device reproducibility, all ultrasound and CT images were resampled to an isotropic voxel resolution of 1 mm³ using trilinear interpolation algorithms. For ROI segmentation, tumor regions of interest were manually delineated on contrast-enhanced arterial phase CT images by the two radiologists, as the arterial phase provides optimal tumor-to-background contrast for accurate boundary definition. Ultrasound images were min-max normalized to the (–1, 1) range, and tumor ROIs were manually delineated by two ultrasonographers using 3D-Slicer (**Supplementary Figure 1**) following standardized protocols. A total of 874 quantitative features were extracted according to Image Biomarker Standardization Initiative (IBSI) guidelines, including morphological (25), first-order statistical (42), texture (729), and wavelet transform features (78) using 8 different wavelet decompositions (LLL, LLH, LHL, LHH, HLL, HLH, HHL, HHH). CT image analysis, independently performed by two blinded imaging physicians, involved spatial standardization using B-spline interpolation, Z-score normalization, and Gaussian filtering (σ=1.0 mm) for noise suppression. To mitigate potential inter-device variability across different scanner manufacturers (Philips, GE, Siemens), intensity harmonization using histogram matching was applied prior to feature extraction. LIFEx software extracted 1433 features following IBSI compliance standards, categorized into morphological (32), first-order statistical (75), texture (620), and filtered transform features (706). The filtered transform features included wavelet decompositions, Laplacian of Gaussian filters, and mathematical transformations (square, square root, logarithm, exponential). For reproducibility analysis of radiomics features, a random subset of 150 patients was selected for repeat feature extraction by the same two operators after a two-week interval to assess intra- and inter-observer reliability. Feature stability across different imaging platforms was assessed through intraclass correlation coefficient (ICC) analysis, with features demonstrating ICC >0.85 retained to ensure cross-device reproducibility.

All radiomics features were standardized using the zero-mean method, with highly correlated features (Spearman’s ρ>0.9) removed. Features with an intraclass correlation coefficient >0.85 were retained to ensure reproducibility.

### Feature selection

T-tests or Mann-Whitney U tests were used to screen features with *P*<0.05 and |log2(Fold Change)|≥1. Subsequently, least absolute shrinkage and selection operator (LASSO) regression was applied to further reduce feature redundancy and optimize feature selection. The optimal λ value was determined using 10-fold cross-validation to identify key predictive features. Correlation heatmaps were generated to analyze relationships between radiomics features and clinical factors, ensuring they could provide complementary information for predicting LLNM.

To identify clinical features associated with LLNM, variance inflation factor (VIF) was calculated through collinearity analysis to exclude variables with multicollinearity. Logistic regression analysis with stepwise regression was then performed on the remaining variables to screen for independent risk factors.

### Multimodal prediction model construction and evaluation

Four machine learning models were constructed: Random Forest (RF), Gradient Boosting Machine (GBM), Support Vector Machine (SVM), and K-Nearest Neighbors (KNN). Models were trained using 10-fold cross-validation to avoid overfitting. While advanced deep learning approaches, including semi-supervised learning frameworks (10) and divide-and-conquer architectures (11), show promise in medical imaging, we selected traditional machine learning algorithms for better interpretability and performance with our dataset size. Hyperparameters were optimized through grid search. Model performance was evaluated by area under the curve (AUC), sensitivity, specificity, accuracy, and related metrics. DeLong tests compared model differences, while clinical utility was assessed using decision curves. SHapley Additive exPlanations (SHAP) and Local Interpretable Model-agnostic Explanations (LIME) were used to interpret model predictions, with SHAP providing global feature importance and LIME offering local explanations for individual cases.

### Statistical analysis

Statistical analyses were performed using R (Version 3.5.3), SPSS (Version 25.0), and Python (Version 3.12.0). Categorical variables were compared using chi-square or Fisher’s exact tests. Continuous variables were compared using t-tests for normally distributed data and Mann-Whitney U tests for non-normally distributed data. Model evaluation included receiver operating characteristic curves, DeLong tests, Decision curve analysis (DCA), and SHAP and LIME analyses. *P*<0.05 was considered statistically significant.

## Results

### Clinical characteristics of patients

In the training group, 146 patients (27.9%) were male and 378 (72.1%) were female, with a mean age of 44.0 ± 12.1 years. The internal validation group consisted of 51 males (22.7%) and 174 females (77.3%), with a mean age of 42.7 ± 11.6 years. The external validation group included 10 males (20.0%) and 40 females (80.0%), with a mean age of 44.2 ± 12.3 years. The incidence of LLNM was 11.6% in the training group, 11.6% in the internal validation group, and 20.0% in the external validation group (**Table 1**). No statistically significant differences in clinical or pathological characteristics were observed between the training and internal validation groups (all *P*>0.05). Follow-up surveillance revealed that 7 patients developed contralateral residual thyroid recurrence. No patients who did not undergo initial lateral neck dissection developed subsequent lateral regional recurrence

**Table 1:** Clinical pathological characteristics of patients.

Clinical features	Training group	Internal validation group	External validation group	t/χ² value^*^	*P* value^*^	t/χ² value^**^	*P* value^**^
Gender							
Male	146 (27.9%)	51 (22.7%)	10 (20.0%)	2.192	0.139	1.347	0.246
Female	378 (72.1%)	174 (77.3%)	40 (80.0%)				
Age (years)	44.0 ± 12.1	42.7 ± 11.6	44.2 ± 12.3	1.365	0.172	0.096	0.924
≥55	97 (18.5%)	38 (16.9%)	8 (16.0%)	0.280	0.596	0.157	0.692
<55	427 (81.5%)	187 (83.1%)	42 (84.0%)				
BMI (kg/m²)	24.26 ± 3.83	24.07 ± 3.76	24.12 ± 3.79	0.626	0.531	0.218	0.827
Normal	317 (60.5%)	144 (64%)	32 (64.0%)	1.067	0.586	0.723	0.697
Overweight	169 (32.3%)	64 (28.4%)	12 (24.0%)				
Obese	38 (7.3%)	17 (7.6%)	6 (12.0%)				
BRAF V600E mutation							
Yes	469 (89.5%)	195 (86.7%)	50 (100.0%)	1.259	0.262	5.837	0.016
No	55 (10.5%)	30 (13.3%)	0 (0.0%)				
Hashimoto’s thyroiditis							
Yes	127 (24.2%)	47 (20.9%)	16 (32.0%)	0.989	0.320	1.126	0.289
No	397 (75.8%)	178 (79.1%)	34 (68.0%)				
Maximum tumor diameter (cm)	1.09 ± 0.79	1.12 ± 0.76	1.03 ± 0.65	0.482	0.630	0.542	0.588
≤1	352 (67.2%)	135 (60.0%)	30 (60.0%)	5.342	0.148	1.234	0.745
>1 to ≤2	121 (23.1%)	68 (30.2%)	16 (32.0%)				
>2 to ≤4	39 (7.4%)	19 (8.4%)	2 (4.0%)				
>4	12 (2.3%)	3 (1.3%)	2 (4.0%)				
Number of lesions							
1	388 (74.0%)	162 (72.0%)	40 (80.0%)	1.579	0.454	0.892	0.345
≥2	136 (26.0%)	63 (28.0%)	10 (20.0%)				
Tumor location							
Upper pole	149 (28.4%)	67 (29.8%)	20 (40.0%)	0.138	0.710	2.147	0.143
Middle/Lower pole	375 (71.6%)	158 (70.2%)	30 (60.0%)				
ETE							
Yes	165 (31.5%)	72 (32.0%)	8 (16.0%)	0.019	0.890	4.325	0.038
No	359 (68.5%)	153 (68.0%)	42 (84.0%)				
Serum thyroglobulin (ng/ml)	34.18 ± 81.86	38.01 ± 80.22	36.37 ± 99.37	0.591	0.555	0.151	0.880
Thyroglobulin antibody (IU/ml)	138.25 ± 466.72	89.31 ± 385.49	275.01 ± 813.53	1.383	0.167	1.127	0.260
Peroxidase antibody (IU/ml)	38.99 ± 88.17	32.33 ± 72.42	76.09 ± 140.89	0.998	0.318	1.674	0.095
White blood cell count (×10^9^/L)	6.80 ± 2.20	6.62 ± 2.20	6.62 ± 2.32	1.027	0.305	0.478	0.633
Platelet count (×10^9^/L)	301.7 ± 95.3	309.7 ± 92.8	287.0 ± 88.5	1.061	0.288	0.947	0.344
Neutrophil count (×10^9^/L)	5.30 ± 1.87	5.41 ± 1.76	5.05 ± 1.70	0.751	0.453	0.822	0.411
Lymphocyte count (×10^9^/L)	1.67 ± 0.81	1.66 ± 0.78	2.02 ± 0.85	0.157	0.876	2.618	0.009
Monocyte count (×10^9^/L)	0.39 ± 0.19	0.36 ± 0.17	0.47 ± 0.14	2.043	0.061	2.763	0.006
Lymphocyte to monocyte ratio	5.54 ± 4.28	5.91 ± 4.38	4.92 ± 3.75	1.077	0.281	0.894	0.372
Neutrophil to lymphocyte ratio	4.21 ± 2.73	4.25 ± 2.65	3.31 ± 2.47	0.185	0.853	2.041	0.042
Platelet to lymphocyte ratio	238.10 ± 146.94	242.69 ± 144.71	186.97 ± 134.17	0.394	0.694	2.146	0.032
SII	1438.7 ± 1187.2	1467.8 ± 1153.0	1066.9 ± 1029.7	0.310	0.756	1.952	0.051
LLNM							
Yes	61 (11.6%)	26 (11.6%)	10 (20.0%)	0.001	0.973	2.354	0.125
No	463 (88.4%)	199 (88.4%)	40 (80.0%)				

*P value for comparison between training group and internal validation group.

**P value for comparison between training group and external validation group.

LLNM, lateral lymph node metastasis; BMI, body mass index; ETE, extrathyroidal extension; SII, Systemic immune inflammation index.

### Clinical risk factors for LLNM

To identify independent risk factors for LLNM, we first performed collinearity diagnostics. Variables demonstrating significant multicollinearity (VIF>10) included platelet count (VIF=99.79), neutrophil count (VIF=89.25), lymphocyte count (VIF=29.22), neutrophil-to-lymphocyte ratio (VIF=178.73), platelet-to-lymphocyte ratio (VIF=160.64), and systemic immune inflammation index (VIF=96.32). These variables were excluded from further analysis to enhance model stability.

Multivariate logistic regression analysis of the remaining variables identified five independent risk factors for predicting LLNM (**Table 2**). Maximum tumor diameter was significantly associated with increased risk, with progressively higher odds for larger tumors (>1 to ≤2 cm: OR=2.494, 95% CI: 1.212–5.132, *P*=0.013; >2 to ≤4 cm: OR=7.851, 95% CI: 3.072–20.066, *P*<0.001; >4 cm: OR=13.032, 95% CI: 3.253–52.212, *P*<0.001). Multiple lesions (≥2 lesions: OR=2.846, 95% CI: 1.436–5.639, *P*=0.003) and tumor location in the upper pole (OR=5.181, 95% CI: 2.550–10.524, *P*<0.001) were also identified as independent risk factors. Additionally, decreased monocyte count (OR=0.004, 95% CI: 0.000–0.070, *P*<0.001) and a lower LMR (OR=0.524, 95% CI: 0.410–0.671, *P*<0.001) were significantly associated with LLNM.

**Table 2:** Collinearity and logistic regression analysis of clinical features associated with LLNM.

Clinical Features	VIF	β	Odds Ratio (95% CI)	*P* value
Gender				
Female			1	
Male	5.41	-0.436	0.646 (0.317-1.317)	0.230
Age (years)				
≥55			1	
<55	1.29	-0.288	0.750 (0.315-1.784)	0.515
BMI (kg/m²)				
Normal			1	
Overweight		0.682	1.977 (0.363-10.766)	0.430
Obese	5.67	-0.374	0.688 (0.119-3.972)	0.676
BRAF V600E mutation				
Yes			1	
No	6.54	-0.511	0.600 (0.223-1.618)	0.313
Hashimoto’s thyroiditis				
No			1	
Yes	2.18	0.293	1.340 (0.547-3.283)	0.522
Maximum tumor diameter (cm)				
≤1			1	
>1 to ≤2		0.914	2.494 (1.212-5.132)	0.013
>2 to ≤4		2.061	7.851 (3.072-20.066)	<0.001
>4	1.72	2.567	13.032 (3.253-52.212)	<0.001
Number of lesions				
1			1	
≥2	5.12	1.046	2.846 (1.436-5.639)	0.003
Tumor location				
Middle/Lower pole			1	
Upper pole	6.95	1.645	5.181 (2.550-10.524)	<0.001
ETE				
Yes			1	
No	1.50	0.731	2.077 (0.997-4.282)	0.058
Serum thyroglobulin (ng/ml)	1.38	-0.001	0.999 (0.996-1.003)	0.753
Thyroglobulin antibody (IU/ml)	1.40	<0.001	1.000 (1.000-1.001)	0.269
Peroxidase antibody (IU/ml)	1.78	0.002	1.002 (0.999-1.005)	0.160
White blood cell count (×10^9^/L)	9.16	0.122	1.129 (0.984-1.297)	0.085
Platelet count (×10^9^/L)	99.79	N/A	N/A	N/A
Neutrophil count (×10^9^/L)	89.25	N/A	N/A	N/A
Lymphocyte count (×10^9^/L)	29.22	N/A	N/A	N/A
Monocyte count (×10^9^/L)	7.26	-5.584	0.004 (0.000-0.070)	<0.001
Lymphocyte to monocyte ratio	7.48	-0.646	0.524 (0.410-0.671)	<0.001
Neutrophil to lymphocyte ratio	178.73	N/A	N/A	N/A
Platelet to lymphocyte ratio	160.64	N/A	N/A	N/A
SII	96.32	N/A	N/A	N/A

LLNM, lateral lymph node metastasis; VIF, Variance inflation factor; BMI, body mass index; ETE, extrathyroidal extension; CI, confidence interval; SII, Systemic immune inflammation index.

### Radiomics feature selection

**Figure 1:**
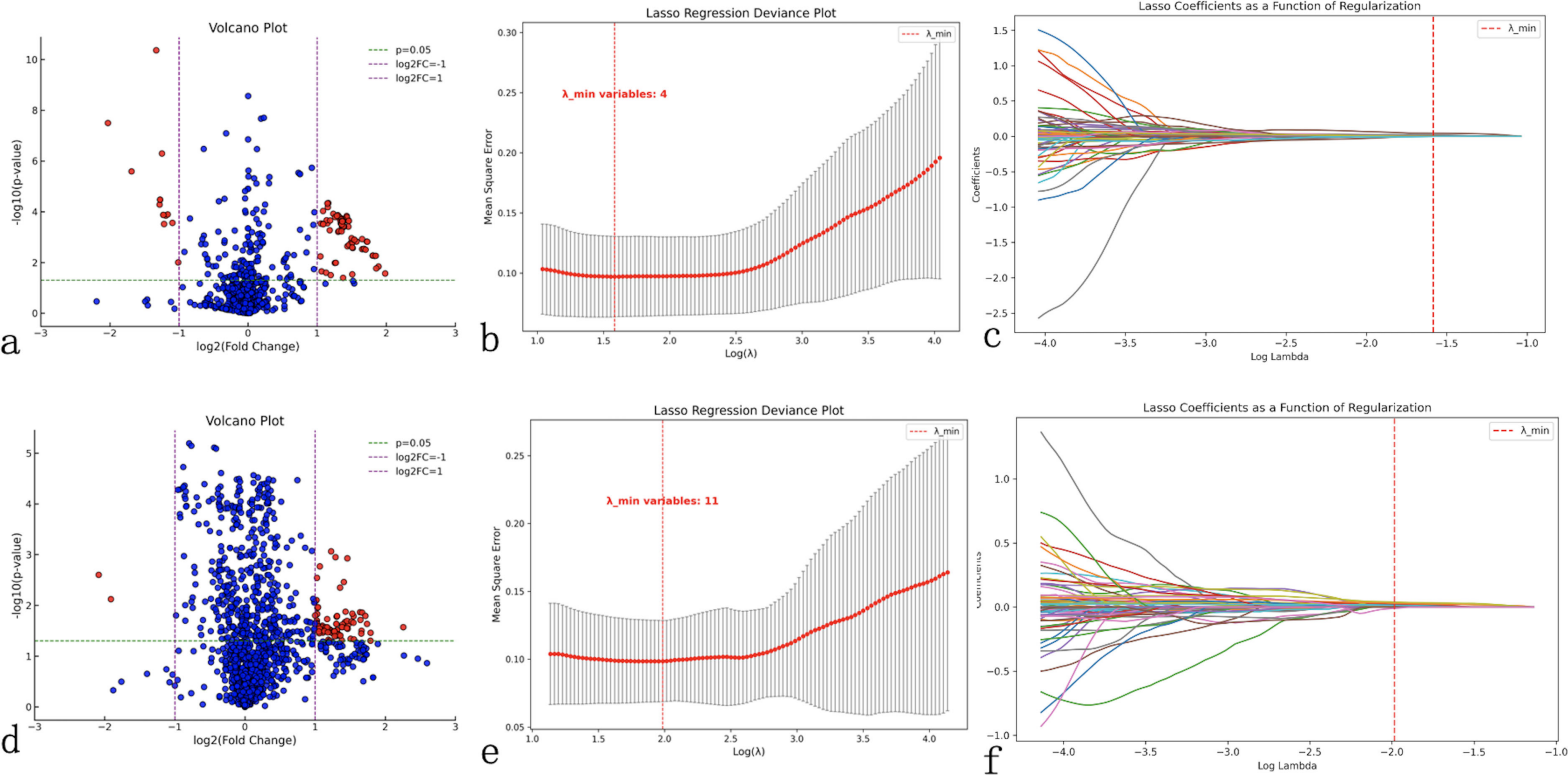
Radiomics feature selection workflow. Comprehensive feature selection process for ultrasound **(a–c)** and computed tomography (CT) radiomics **(d–f)**. **(a, d)** Volcano plots display the relationship between statistical significance (-log10(p-value), y-axis) and fold change magnitude (log2(fold change), x-axis) for all extracted features, with red dots indicating statistically significant features (*P*<0.05, |log2(fold change)|≥1) selected for further analysis. **(b, e)** Least Absolute Shrinkage and Selection Operator (LASSO) regression deviance plots showing cross-validation error (mean-squared error, y-axis) versus regularization parameter lambda (log(λ), x-axis), with the optimal lambda value (red dashed line) minimizing prediction error while reducing feature redundancy. **(c, f)** LASSO coefficient plots demonstrating feature selection process, where each colored line represents a radiomics feature’s coefficient value changing with regularization strength, with features retained at optimal lambda shown as non-zero coefficients. This process reduced 874 ultrasound features to 4 key predictors and 1433 CT features to 11 key predictors for model construction.

Differential feature analysis using independent sample t-tests (for normally distributed data) or Mann-Whitney U tests (for skewed distributions) was performed to compare radiomics features between groups with and without LLNM. Features with *P*<0.05 and |log2(Fold Change)|≥1 were selected. This initial screening reduced the 874 ultrasound radiomics features to 100 and the 1433 CT radiomics features to 97 (**Figures 1a**).

LASSO regression was subsequently applied to further eliminate redundant features while balancing model simplicity and predictive performance. Using 10-fold cross-validation to determine the optimal λ value (corresponding to minimum binomial deviance), we identified 4 key ultrasound features (1 first-order feature and 3 texture features) and 11 key CT features (1 morphological feature and 10 texture features) (**Figures 1b**).

**Figure 2:**
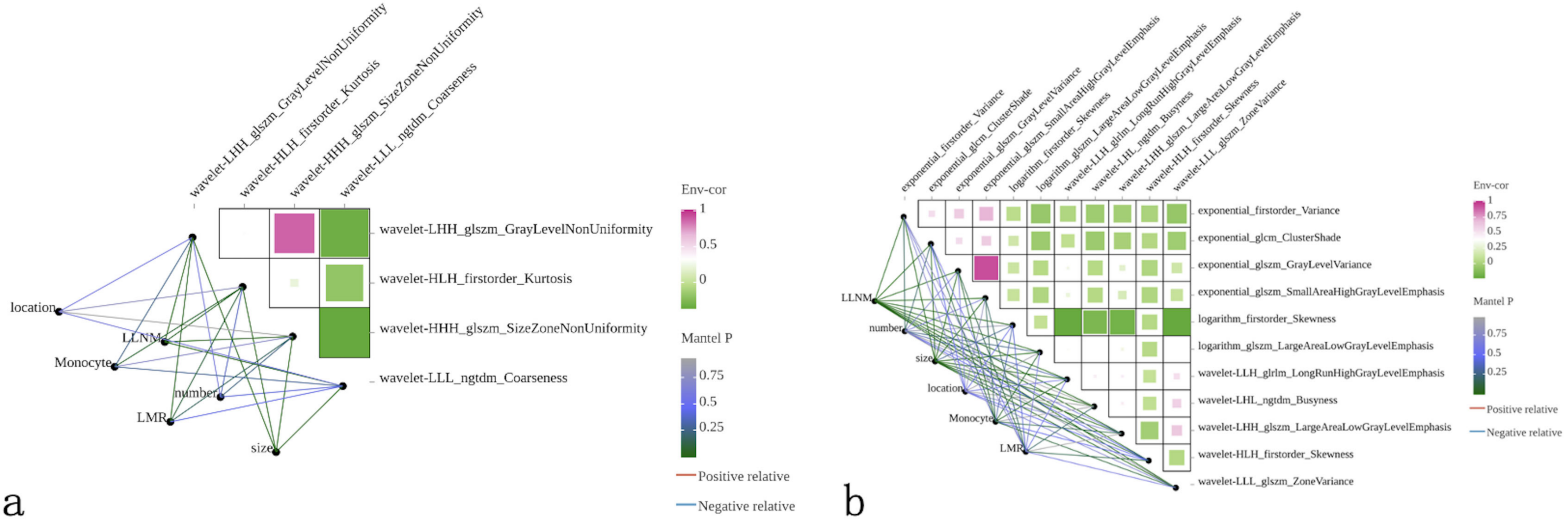
Correlation analysis between radiomics features and clinical variables. Heatmaps displaying Pearson correlation coefficients between selected radiomics features and clinical factors for **(a)** ultrasound and **(b)** CT modalities. Color intensity represents correlation strength, with green indicating positive correlations and purple indicating negative correlations. Clinical variables include tumor location, lateral lymph node metastasis (LLNM) status, monocyte count, lymphocyte-to-monocyte ratio (LMR), lesion number, and tumor size. Network diagrams show interconnections between variables, with line thickness representing correlation strength. Weak correlations between radiomics features and clinical factors (except LLNM) demonstrate that imaging-derived features provide complementary information to traditional clinical parameters, supporting the rationale for multimodal model development.

Correlation analysis using heatmaps evaluated the relationships between radiomics features and clinical factors (tumor diameter, number of lesions, tumor location, monocyte count, LMR and LLNM). Results demonstrated weak correlations between clinical factors, except for LLNM, and radiomics features (**Figures 2a**), indicating that these features could provide complementary information for constructing more robust prediction models.

### Construction of multimodal machine learning models

Based on the 5 clinical features, 4 ultrasound radiomics features, and 11 CT radiomics features, we constructed four machine learning models: RF, GBM, SVM, and KNN. Comprehensive evaluation revealed that the GBM model demonstrated superior overall performance, achieving AUCs of 0.973, 0.803, and 0.975 in the training, internal validation, and external validation sets, respectively (**Table 3**).

**Table 3:** Performance of four models in training, internal validation and external validation sets.

	AUC	AUC 95% CI	Accuracy	Specificity	Sensitivity	PPV	NPV	F1 score	Loss value	AP
Training										
RF	0.924	0.895-0.953	0.943	0.957	0.913	0.913	0.957	0.913	0.269	0.896
GBM	0.973	0.956-0.987	0.914	0.894	0.957	0.815	0.977	0.880	0.300	0.952
SVM	0.932	0.905-0.959	0.957	0.957	0.957	0.917	0.978	0.936	0.183	0.915
KNN	0.914	0.885-0.943	0.786	0.702	0.957	0.611	0.971	0.746	0.367	0.872
Internal validation										
RF	0.811	0.745-0.877	0.755	0.849	0.866	0.651	0.797	0.605	0.493	0.795
GBM	0.803	0.735-0.871	0.725	0.889	0.794	0.639	0.747	0.487	1.720	0.785
SVM	0.715	0.635-0.795	0.681	0.819	0.404	0.526	0.734	0.457	0.867	0.658
KNN	0.744	0.670-0.818	0.725	0.769	0.636	0.578	0.810	0.606	1.825	0.728
External validation										
RF	0.955	0.925-0.978	0.900	0.850	1.000	0.769	1.000	0.870	0.439	0.938
GBM	0.975	0.933-0.982	0.900	0.950	0.800	0.889	0.905	0.842	0.364	0.961
SVM	0.910	0.825-0.995	0.800	0.850	0.700	0.700	0.850	0.700	0.338	0.872
KNN	0.713	0.675-0.851	0.567	0.500	0.700	0.412	0.769	0.519	1.853	0.684

*AUC*, Area Under the Curve; *CI*, Confidence Interval; *PPV*, Positive Predictive Value; *NPV*, Negative Predictive Value; *AP*, Average Precision; *RF*, Random Forest; *GBM*, Gradient Boosting Machine; *SVM*, Support Vector Machine; *KNN*, K-Nearest Neighbors.

**Figure 3:**
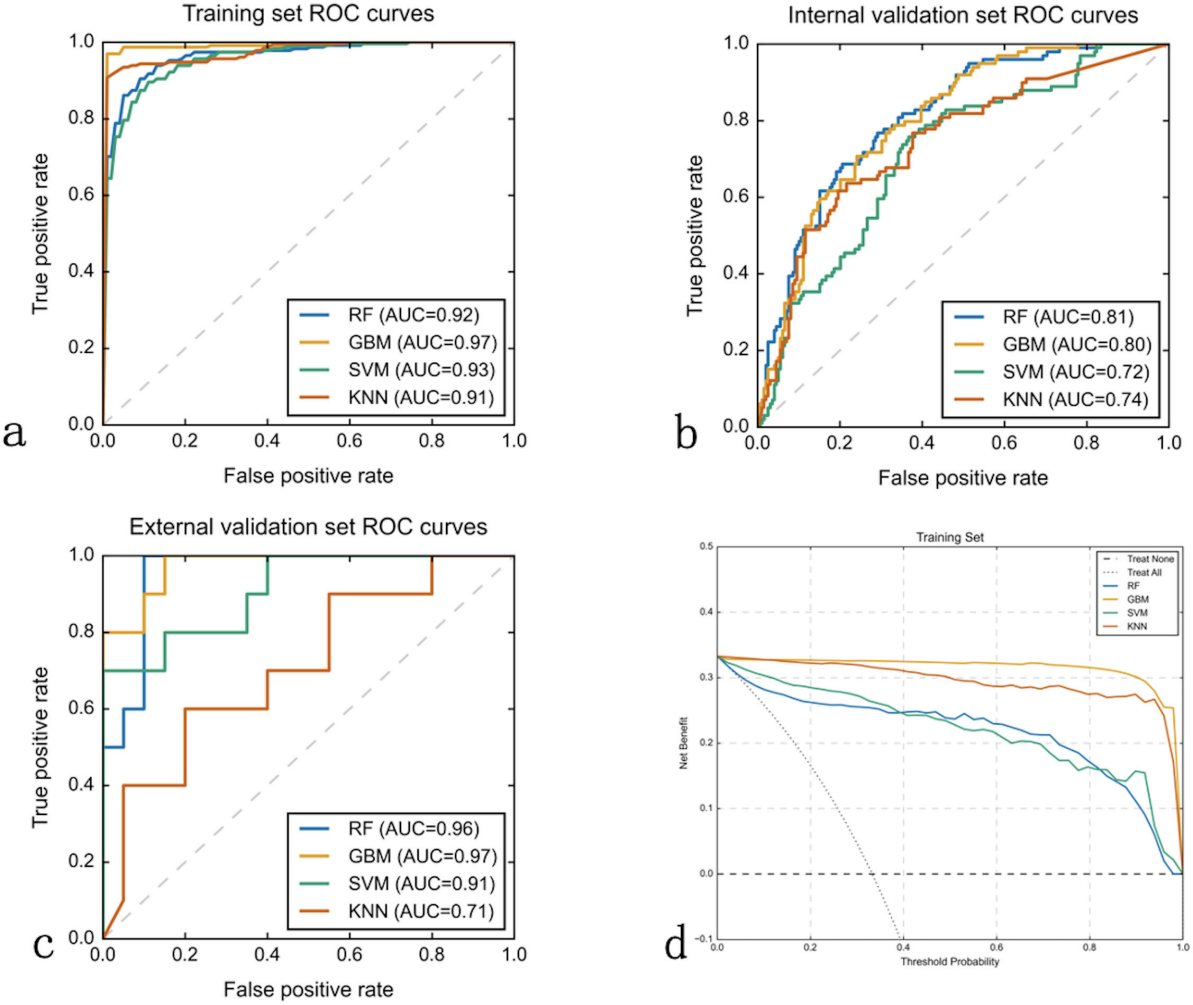
Machine learning model performance comparison. Receiver Operating Characteristic (ROC) curves and decision curve analysis (DCA) evaluating four machine learning algorithms across three datasets. **(a–c)** ROC curves plot true positive rate (sensitivity, y-axis) versus false positive rate (1-specificity, x-axis) for Random Forest (RF), Gradient Boosting Machine (GBM), Support Vector Machine (SVM), and K-Nearest Neighbors (KNN) models in training **(a)**, internal validation **(b)**, and external validation **(c)** sets. Area Under the Curve (AUC) values quantify discriminative performance. GBM consistently achieved superior performance with AUCs of 0.97, 0.80, and 0.97 respectively. **(d)** Decision curve analysis for the training set displays net benefit (y-axis) versus threshold probability (x-axis), with GBM (yellow line) providing highest clinical utility across all probability thresholds compared to treating all patients (horizontal line) or no treatment (diagonal line).

In the training set, the GBM model achieved an accuracy of 0.914, with 0.894 specificity and 0.957 sensitivity. In the internal validation set, these metrics were 0.725, 0.889, and 0.794, respectively. In the external validation set, the GBM model demonstrated excellent performance with an accuracy of 0.900, specificity of 0.950, and sensitivity of 0.800 (**Table 3**). Although the RF model showed a comparable AUC in the external validation set (0.955 *vs*. 0.975), the GBM model exhibited better overall diagnostic metrics and F1 score (0.842 *vs*. 0.870). DeLong tests revealed statistically significant differences between the GBM model’s AUC and other models (*P*<0.05) (**Figures 3a**). Combined with the higher AUC values, this indicates that the GBM model achieved superior discriminative performance compared to the RF, SVM, and KNN models. DCA further validated these findings: across clinically relevant threshold ranges, the GBM model (yellow line) maintained the highest net benefit, followed by KNN, SVM, and RF models (**Figure 3**).

**Figure 4:**
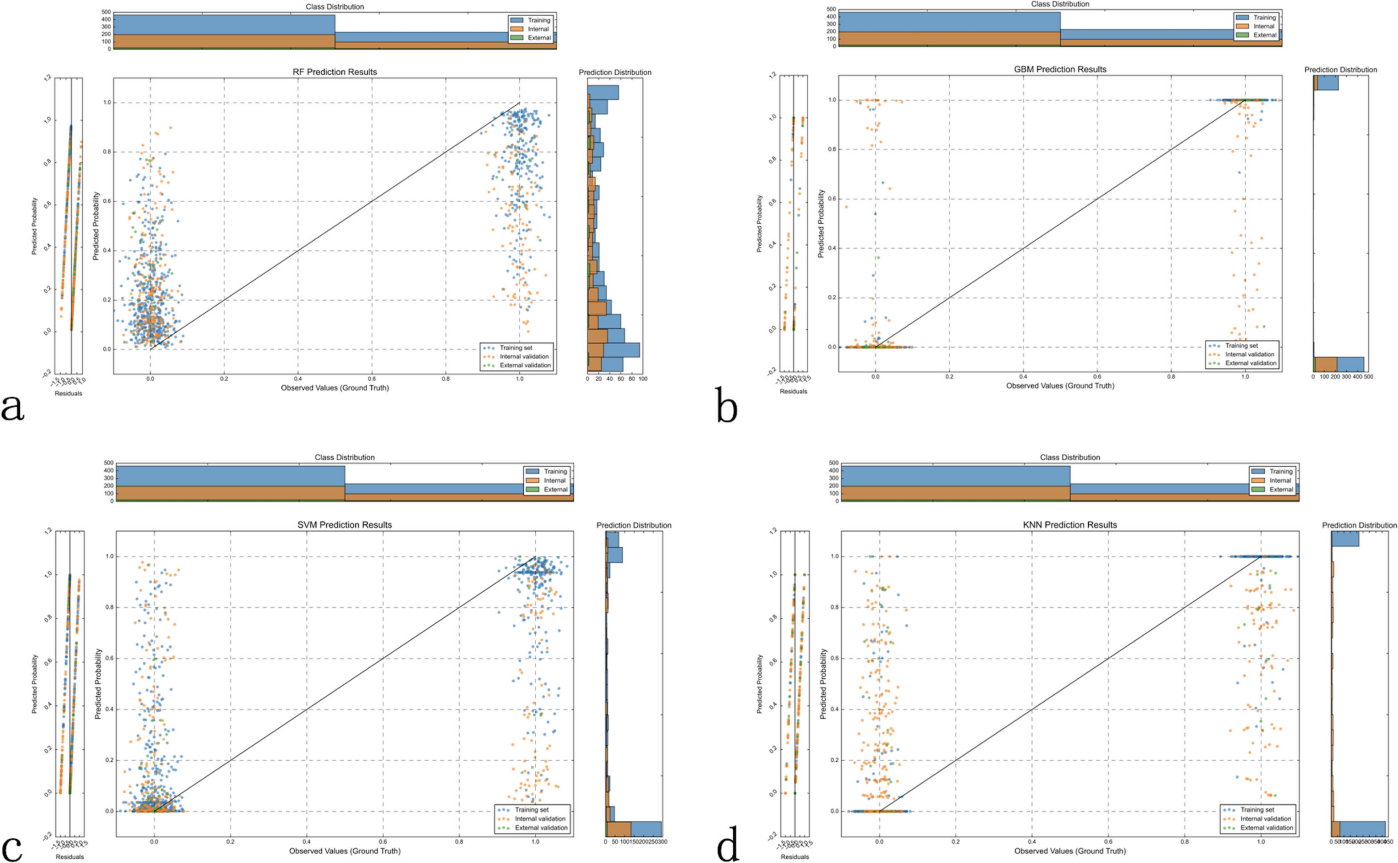
Model prediction probability distribution analysis. Scatter plots displaying the distribution of predicted probabilities (y-axis) versus observed outcomes (x-axis, where 0 = no LLNM, 1 = LLNM) across all three datasets for each machine learning model. Optimal model performance shows low predicted probabilities clustered near y=0 for patients without LLNM (x=0) and high predicted probabilities clustered near y=1 for patients with LLNM (x=1). **(a)** Random Forest (RF) demonstrates moderate separation between the two outcome groups, with some overlap in predicted probabilities between LLNM-positive and LLNM-negative cases. **(b)** Gradient Boosting Machine (GBM) shows the clearest separation between outcome groups, with LLNM-negative cases predominantly clustered at low predicted probabilities and LLNM-positive cases at high predicted probabilities, indicating superior discriminative ability. **(c)** Support Vector Machine (SVM) exhibits less distinct separation, with notable prediction overlap between the two outcome groups, particularly affecting discrimination accuracy. **(d)** K-Nearest Neighbors (KNN) shows considerable overlap between outcome groups, reflecting limited discriminative capability. Histograms display probability density distributions for each class (blue = no LLNM, orange = LLNM), with better models showing more distinct, non-overlapping distributions. GBM’s superior class separation and minimal probability overlap support its selection as the optimal prediction model.

The observation-prediction probability scatter plots revealed the predictive characteristics of each model (**Figures 4a**). The RF model (**Figure 4**) exhibits a characteristic “striped” pattern due to its ensemble voting mechanism, with good overall separation but notable uncertainty in the mid-probability range. The GBM model (**Figure 4**) demonstrates a more continuous probability distribution with clearer class separation, reflecting its superior calibration and generalization capability. The SVM model (**Figure 4**) displays a polarized prediction pattern, clustering probabilities at extremes, which corresponds to its lower sensitivity (0.404) in internal validation due to misclassifications, particularly in the external validation set. The KNN model (**Figure 4**) produces a “stepped” probability pattern, indicating limited discrimination ability and aligning with its lower accuracy (0.567) and F1 score (0.519) in external validation. These probability distributions visually corroborate the performance metrics in **Table 3**, further supporting the GBM model as the most reliable classifier for LLNM prediction.

### Feature importance and model interpretation using SHAP and LIME

**Figure 5:**
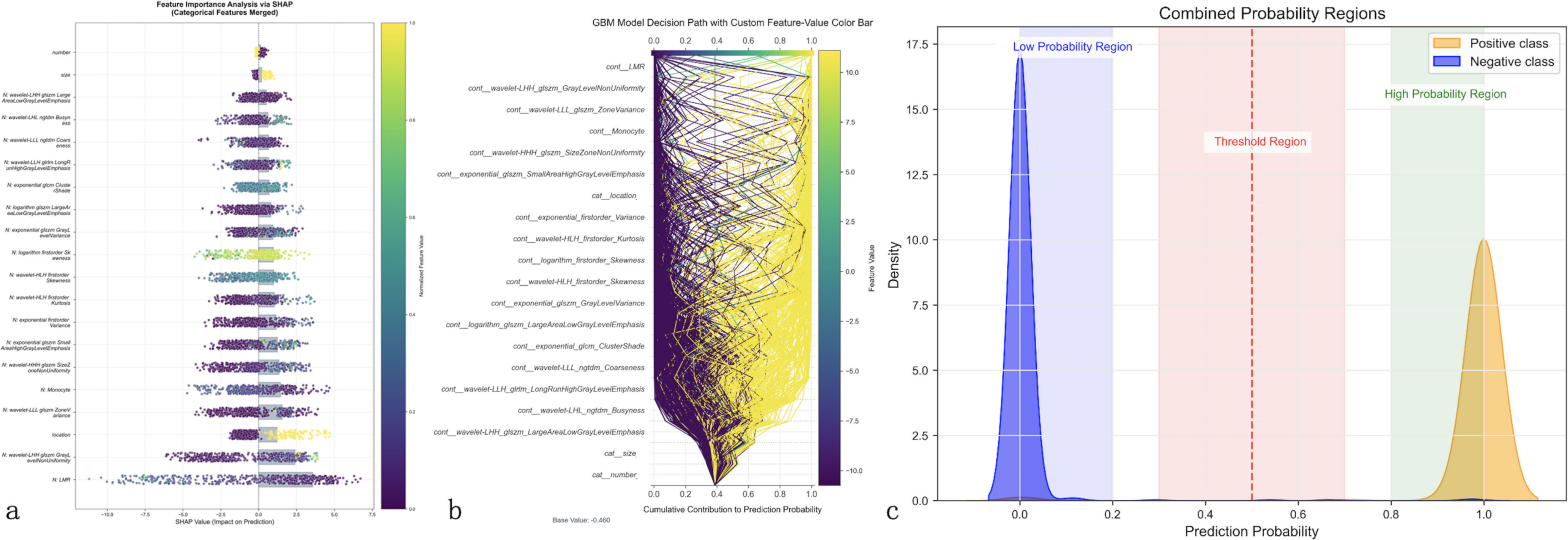
Model interpretability analysis using advanced explainable AI techniques. Comprehensive feature importance analysis of the optimal GBM model using SHapley Additive exPlanations (SHAP) and Local Interpretable Model-agnostic Explanations (LIME). **(a)** SHAP summary plot ranks features by importance (y-axis) with individual patient predictions shown as colored dots, where color intensity represents feature values (purple = low, yellow = high) and horizontal position indicates impact on prediction (leftward = decreased LLNM risk, rightward = increased risk). Lymphocyte-to-monocyte ratio (LMR) emerges as the most influential predictor. **(b)** SHAP decision path plot illustrates cumulative feature contributions from baseline probability (0.4) to final predictions, with each line representing an individual patient’s prediction pathway, demonstrating complex feature interactions. **(c)** LIME probability distribution analysis shows clear class separation with minimal overlap between positive (orange) and negative (blue) cases, confirming robust risk stratification capability across the probability spectrum.

SHAP analysis was employed to interpret the GBM model’s prediction process. SHAP analysis (**Figure 5**) identifies LMR as the most influential feature, followed by wavelet-based texture features (wavelet-LHH_glszm_GrayLevelNonUniformity, wavelet-LLL_glszm_ZoneVariance), morphological characteristics (wavelet-HHH_glszm_SizeZoneNonUniformity), and exponential transformations of gray-level matrices. The color gradient highlights how feature values impact prediction outcomes. **Figure 5** visualizes the model’s decision path, mapping cumulative feature contributions from the base value (0.4) to final probabilities (0–1), revealing complex feature interactions with stronger positive contributions toward LLNM prediction.

LIME analysis (**Figure 5**) demonstrates clear probability stratification, with distinct low- and high-risk regions and a minimal overlap between positive and negative cases, supporting robust risk discrimination. These findings indicate that while LMR is the dominant predictor, the GBM model’s superior performance arises from the integration of clinical and radiomic features, enhancing LLNM prediction beyond conventional visual assessment.

### Clinical implementation of the prediction model

**Figure 6:**
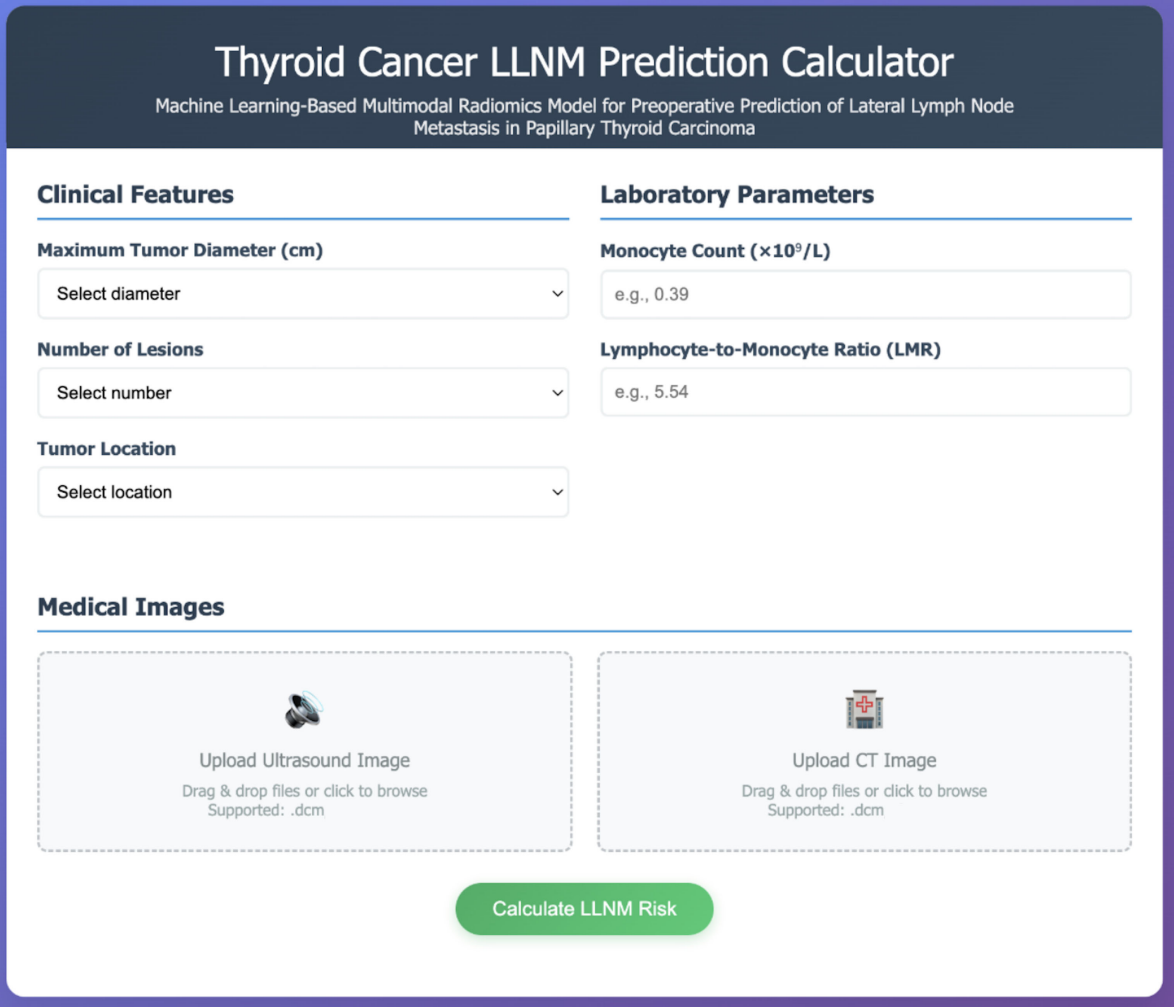
Web-based clinical implementation interface. Interactive prediction calculator implementing the validated GBM multimodal model for real-time clinical decision support. The interface integrates three essential components: (1) Clinical Features section with dropdown menus for maximum tumor diameter, number of lesions, and tumor location; (2) Laboratory Parameters section with numerical input fields for monocyte count (×10^9^/L) and lymphocyte-to-monocyte ratio (LMR), including example values for guidance; (3) Medical Images section with drag-and-drop functionality supporting DICOM format uploads for both ultrasound and CT images, enabling automated radiomics feature extraction. The “Calculate LLNM Risk” button processes all inputs through the trained model to provide instantaneous probability assessment with clinical recommendations, facilitating evidence-based surgical planning and reducing diagnostic uncertainty in papillary thyroid carcinoma management.

To facilitate clinical translation of our multimodal prediction model, we developed an interactive web-based calculator interface (**Figure 6**). This platform integrates all identified predictive features into a streamlined clinical workflow, including: (a) dropdown menus for clinical features (tumor diameter, lesion number, tumor location); (b) input fields for laboratory parameters (monocyte count, LMR); (c) DICOM image upload functionality for ultrasound and CT images; and (d) real-time LLNM probability calculation. This implementation provides healthcare providers with a practical tool for preoperative risk stratification in PTC patients.

## Discussion

Despite technological advances, preoperative LLNM diagnosis remains challenging due to significant technical and interpretative limitations. Current methods rely heavily on subjective radiologist interpretation of imaging features with inherent variability. Standard ultrasound criteria and CT assessment demonstrate only moderate sensitivity and insufficient specificity (3, 12), particularly for micrometastases smaller than 2mm that cause minimal morphological changes. Even experienced radiologists struggle to differentiate reactive lymph nodes from early metastatic involvement due to overlapping features. This diagnostic gap significantly impacts surgical decision-making, forcing surgeons to balance the risks of potentially unnecessary therapeutic lateral neck dissection against the oncological consequences of leaving occult metastases untreated. These persistent challenges underscore the urgent need for more objective, quantitative approaches to preoperative LLNM risk assessment. The variability in LLNM prevalence across different institutions, as evidenced by the higher rate observed in our external validation cohort (20.0%) compared to the training cohort (11.6%), further highlights the complexity of standardizing diagnostic approaches across diverse clinical settings with varying referral patterns and case complexities.

Previous studies on LLNM prediction have primarily relied on either clinical parameters or single-modality imaging analysis. Several notable imaging-based efforts include Zou et al.’s combined dual-energy CT and thyroid function indicators model (AUC: 0.834 in the full cohort) (13), Jiang et al.’s contrast-enhanced ultrasound-based radiomics nomogram (AUC: 0.820 in training set) (14), and other recent advances that have demonstrated promising results with CT radiomics-based approaches. These include prospective multicenter studies achieving robust performance in lateral neck lymph node metastasis prediction (15), as well as specialized models targeting challenging cases such as lymph nodes with short diameter less than 8mm (16). Other researchers have focused on developing prediction models based solely on clinical risk factors like tumor size, age, gender, and conventional laboratory parameters (1, 17). Despite these promising results, these approaches have inherent limitations: they typically utilize either clinical parameters or single imaging modality features without leveraging the complementary information available from integrating multiple data sources. Additionally, most existing models operate as diagnostic “black boxes” without clear explanations of their decision-making process, and the absence of external validation in many studies restricts their generalizability to diverse clinical settings (18).

Our multimodal machine learning approach addresses these limitations by seamlessly integrating clinical characteristics with both ultrasound and CT radiomics features. The GBM model demonstrated superior performance across all datasets, with AUCs of 0.973, 0.803, and 0.975 in the training, internal validation, and external validation sets, respectively. The notable decrease in AUC from training to internal validation (0.973 to 0.803) may reflect inherent data heterogeneity within the single-center population and natural variations in sample composition between cohorts. This performance variation, while indicating opportunities for further optimization through enhanced feature selection strategies, more rigorous cross-validation approaches and improved model calibration techniques, still resulted in superior performance compared to other approaches. This performance significantly outpaced other machine learning algorithms, including RF, SVM, and KNN models. The GBM model’s excellent generalization capability was evidenced by its evenly distributed prediction probabilities across the entire range, as shown in **Figure 3**, in contrast to the more fragmented patterns exhibited by other models. Importantly, despite the external validation cohort’s substantially higher LLNM prevalence (20.0% *vs* 11.6%), our model maintained robust performance (AUC: 0.975), demonstrating resilience to population heterogeneity and case-mix variations that commonly occur across different clinical settings. Unlike most previous work, our approach incorporates advanced interpretability techniques—SHAP and LIME—transforming the typically opaque machine learning model into a transparent, explainable system (19). This interpretability enhances clinical trust and facilitates understanding of the model’s predictions, addressing a key barrier to clinical implementation of artificial intelligence systems in healthcare. Recent studies have similarly emphasized the importance of explainable machine learning approaches in predicting lymph node metastasis in thyroid cancer, demonstrating the broader clinical acceptance and applicability of interpretable AI in oncological decision-making (20).

Our SHAP analysis identified LMR as the most influential predictor for LLNM, consistent with established research on immune microenvironment’s role in tumor metastasis. This finding underscores the biological significance of immune parameters in metastasis development (21). In contrast to recent studies showing sex and age as significant predictors of lymph node metastasis in PTC (22), these traditional demographic factors did not reach statistical significance in our LLNM prediction model, highlighting the distinct predictive patterns for lateral versus central lymph node metastasis. Lymphocytes display dual regulatory properties—effector cells provide anti-tumor immunity while tumor cells recruit immunosuppressive T-regulatory cells to facilitate immune evasion (23). Monocytes contribute significantly by differentiating into tumor-associated macrophages that promote angiogenesis and metastatic spread (24). The LMR serves as a quantifiable indicator of this immunological balance, with lower values potentially reflecting both diminished anti-tumor surveillance and enhanced pro-tumorigenic processes (25, 26). The key radiomics features selected by our model complement these immune indicators by capturing tumor heterogeneity and invasive behavior at the microstructural level. To enhance clinical understanding, we provide detailed biological interpretations of these top-ranked radiomic features. Specifically, the ultrasound-derived wavelet-LHH_glszm_GrayLevelNonUniformity captures high-frequency spatial variations in image intensity, reflecting internal tumor architecture and cellular disorganization associated with invasive growth (27). Higher values indicate greater intratumoral heterogeneity, suggesting regions of variable cellular density, necrosis, or vascular changes associated with metastatic capability. The CT-derived wavelet-LLL_glszm_ZoneVariance quantifies low-frequency texture variations, representing larger-scale structural patterns within the tumor that characterize tumor density and boundary properties indicative of matrix remodeling and active invasion fronts (28, 29). Additionally, the wavelet-HHH_glszm_SizeZoneNonUniformity measures variation in connected region sizes at high frequencies, indicating irregular tumor borders and infiltrative growth patterns typical of metastatic lesions (30). These radiomic signatures capture subtle microstructural changes invisible to conventional visual assessment, providing quantitative biomarkers of tumor biology that complement traditional clinical and laboratory parameters in predicting LLNM risk. LIME analysis further validated our model’s robust discriminative ability, demonstrating clear probability stratification with minimal overlap between positive and negative cases. Together, these findings represent digital signatures of biological processes typically invisible to conventional visual assessment (31).

Our multimodal prediction model offers substantial clinical value through several mechanisms. As a non-invasive preoperative risk stratification tool, it enables more informed surgical planning regarding lateral neck dissection. For patients identified as high-risk of LLNM, clinicians can implement more comprehensive evaluation with targeted ultrasound by experienced sonographers or additional imaging modalities such as contrast-enhanced thin-slice CT, potentially reducing both unnecessary lateral neck dissections in low-risk patients and missed metastases requiring secondary surgery in high-risk individuals (32). For active surveillance candidates, the model provides valuable additional information to inform treatment decisions. The model’s robust performance in the external validation cohort, which exhibited a markedly different LLNM prevalence pattern potentially reflecting institutional differences in referral practices or patient demographics, demonstrates meaningful adaptability to varying clinical contexts that characterize real-world healthcare environments. This cross-institutional validation under heterogeneous conditions enhances confidence in the model’s potential for widespread implementation across diverse medical centers. Additionally, the interpretability through SHAP and LIME analyses gives clinicians transparent insights into specific factors contributing to individual risk profiles, facilitating more personalized patient counseling and treatment planning (33).

To facilitate widespread clinical adoption of our GBM-based prediction model, we have designed a user-friendly web-based calculator (**Figure 6**) that addresses radiomics infrastructure limitations. This online platform allows clinicians to upload standard DICOM ultrasound and CT images through a secure web interface, where automated algorithms process the images and compute radiomics features without requiring local technical expertise. The web calculator interface is designed for clinical efficiency, with an estimated completion time of 3–5 minutes for data input and image upload, followed by automated processing within 2–3 minutes. Users input basic clinical parameters (tumor diameter, lesion number, location, monocyte count, and LMR) and upload corresponding images. While the current implementation requires manual parameter entry and image upload, future integration with hospital Picture Archiving and Communication Systems could significantly streamline the workflow by automatically retrieving patient imaging data and laboratory results from electronic health records. The system automatically performs image preprocessing and feature extraction using our validated algorithms, generating a comprehensive risk assessment report with predicted LLNM probability and clinical recommendations within minutes.

Despite its strengths, our study has several limitations. First, as a retrospective study, potential selection bias cannot be completely eliminated, and patient allocation was not randomized across centers. Second, radiomics feature extraction and analysis methods lack full standardization across institutions, potentially affecting reproducibility and clinical translation (34). The imaging protocols, while standardized within each center, may vary between institutions, introducing technical variability. Third, our model currently incorporates ultrasound and CT radiomics but could benefit from additional imaging modalities such as contrast-enhanced ultrasound, MRI, or molecular imaging techniques (35–37) to further enhance predictive accuracy. Finally, the external validation cohort (n=50) represents a significant limitation that restricts comprehensive assessment of model generalizability across broader population demographics. This sample size is insufficient for robust statistical evaluation of model performance variability under different institutional characteristics and may not adequately represent the full spectrum of real-world clinical heterogeneity encountered in diverse healthcare systems. Future validation should include: (1) prospective multicenter studies involving 5–8 tertiary centers with 300–500 patients to ensure adequate statistical power; (2) international validation across different healthcare systems to assess model transferability; (3) temporal validation using consecutive patient cohorts to evaluate model stability; and (4) equipment diversity validation across different imaging platforms to assess feature reproducibility. To address these limitations, future research priorities should focus on large-scale prospective multicenter validation, standardization of imaging acquisition and processing workflows, and incorporation of emerging imaging technologies to establish robust clinical implementation guidelines.

## Conclusions

In summary, our study integrated clinical features, ultrasound radiomics, and CT radiomics data to construct a multimodal model for predicting LLNM in PTC patients using machine learning algorithms. The model demonstrated excellent predictive performance and clinical application potential, providing an objective basis for individualized precision treatment of PTC. By enabling more accurate preoperative risk stratification, this approach may reduce missed metastases requiring secondary surgery, ultimately improving patient outcomes through more personalized surgical management.

## Data Availability

The raw data supporting the conclusions of this article will be made available by the authors, without undue reservation.

## Supplementary material

Supplementary Figure 1 Multimodal Medical Imaging Workflow for Radiomics Analysis. Step-by-step illustration of the imaging analysis pipeline demonstrating tumor segmentation and feature extraction methodology. **(a)** Original high-resolution ultrasound image showing thyroid tumor with characteristic echogenic patterns and surrounding normal thyroid tissue. **(b)** Same ultrasound image with manual region of interest (ROI) delineation overlaid in green, performed by experienced ultrasonographers using 3D-Slicer software following standardized protocols to ensure reproducible tumor boundary definition. **(c)** Corresponding axial CT image at the same anatomical level with precisely matched ROI segmentation (green overlay), enabling cross-modal feature correlation and multimodal analysis. **(d)** Three-dimensional volumetric reconstruction of the segmented tumor volume, providing comprehensive spatial representation for advanced radiomics feature extraction including morphological, textural, and transform-based parameters.
