# Supplementary figures and images for: Application and validation of the machine learning-based multimodal radiomics model for preoperative prediction of lateral lymph node metastasis in papillary thyroid carcinoma
Source: Front Endocrinol (Lausanne). 2025 Aug 19;16:1618902. doi: 10.3389/fendo.2025.1618902 (PMC12401691; doi:10.3389/fendo.2025.1618902)

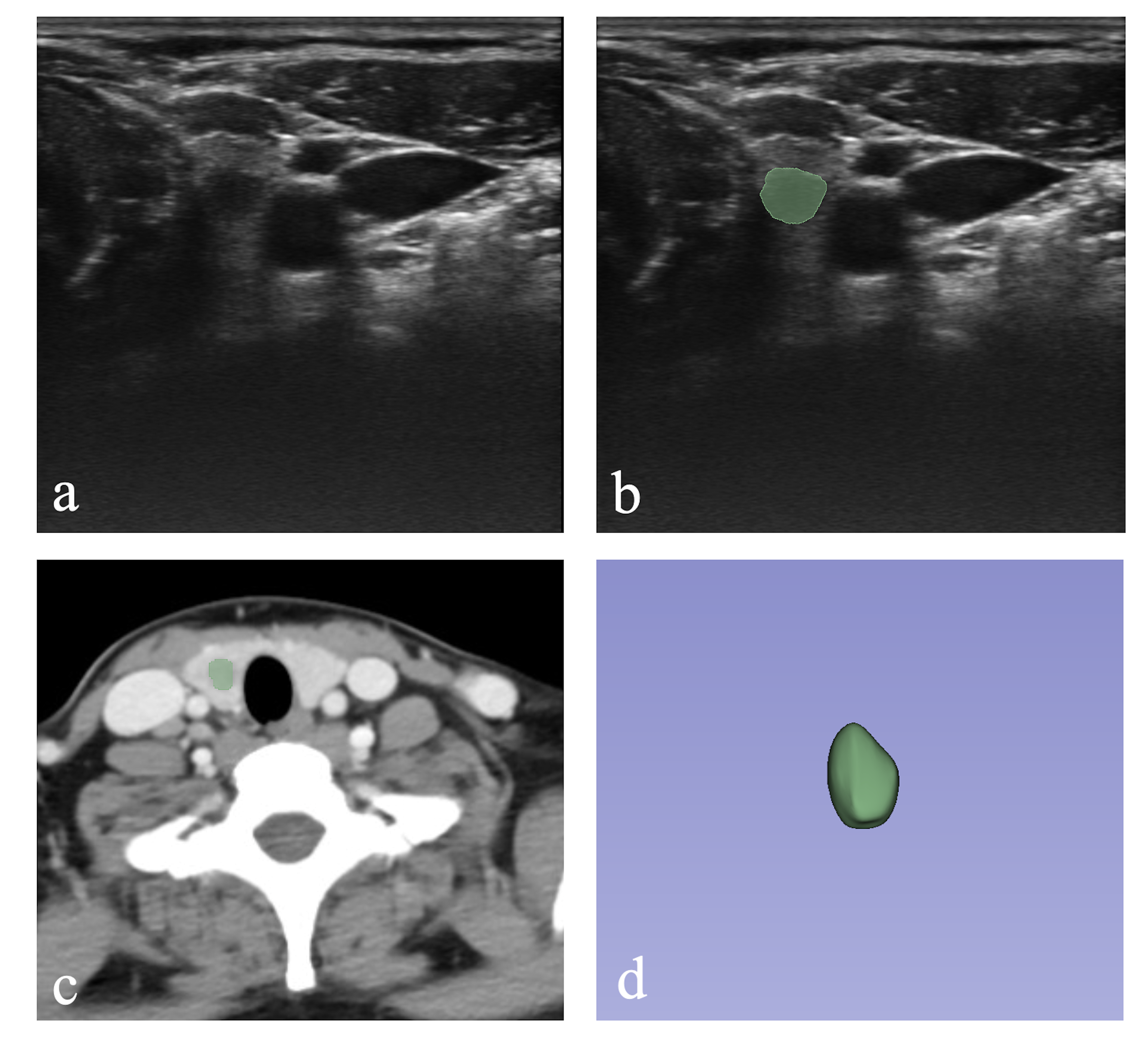

Supplement: Supplementary Figure 1 — Multimodal Medical Imaging Workflow for Radiomics Analysis. Step-by-step illustration of the imaging analysis pipeline demonstrating tumor segmentation and feature extraction methodology. (a) Original high-resolution ultrasound image showing thyroid tumor with characteristic echogenic patterns and surrounding normal thyroid tissue. (b) Same ultrasound image with manual region of interest (ROI) delineation overlaid in green, performed by experienced ultrasonographers using 3D-Slicer software following standardized protocols to ensure reproducible tumor boundary definition. (c) Corresponding axial CT image at the same anatomical level with precisely matched ROI segmentation (green overlay), enabling cross-modal feature correlation and multimodal analysis. (d) Three-dimensional volumetric reconstruction of the segmented tumor volume, providing comprehensive spatial representation for advanced radiomics feature extraction including morphological, textural, and transform-based parameters. [file Image1.tiff]
